# Development and evaluation of two whole-blood flow cytometry protocols for monitoring patients treated with JAK inhibitors

**DOI:** 10.1093/immadv/ltaf006

**Published:** 2025-03-12

**Authors:** Louis Waeckel, Chloé Talon, Mathilde Barrau, Anne-Emmanuelle Berger, Xavier Roblin, Stéphane Paul

**Affiliations:** Immunology Laboratory, iBiothera Reference Center, CHU Saint-Etienne, F42055 Saint-Etienne, France; CIRI - Centre International de Recherche en Infectiologie, Team GIMAP, Université Claude Bernard Lyon 1, Inserm, U1111, CNRS, UMR5308, F42023 Saint-Etienne, France; CIC 1408 Inserm Vaccinology, CHU Saint-Etienne, F42055 Saint-Etienne, France; Immunology Laboratory, iBiothera Reference Center, CHU Saint-Etienne, F42055 Saint-Etienne, France; Department of Gastroenterology, CHU Saint-Etienne, Saint-Etienne, France; Immunology Laboratory, iBiothera Reference Center, CHU Saint-Etienne, F42055 Saint-Etienne, France; CIRI - Centre International de Recherche en Infectiologie, Team GIMAP, Université Claude Bernard Lyon 1, Inserm, U1111, CNRS, UMR5308, F42023 Saint-Etienne, France; CIC 1408 Inserm Vaccinology, CHU Saint-Etienne, F42055 Saint-Etienne, France; CIRI - Centre International de Recherche en Infectiologie, Team GIMAP, Université Claude Bernard Lyon 1, Inserm, U1111, CNRS, UMR5308, F42023 Saint-Etienne, France; CIC 1408 Inserm Vaccinology, CHU Saint-Etienne, F42055 Saint-Etienne, France; Department of Gastroenterology, CHU Saint-Etienne, Saint-Etienne, France; Immunology Laboratory, iBiothera Reference Center, CHU Saint-Etienne, F42055 Saint-Etienne, France; CIRI - Centre International de Recherche en Infectiologie, Team GIMAP, Université Claude Bernard Lyon 1, Inserm, U1111, CNRS, UMR5308, F42023 Saint-Etienne, France; CIC 1408 Inserm Vaccinology, CHU Saint-Etienne, F42055 Saint-Etienne, France

**Keywords:** JAK inhibitors, flow cytometry, STAT, cytokines, therapeutic drug monitoring

## Abstract

**Introduction:**

The clinical efficacy of Janus kinase inhibitors (JAKinibs) is highly variable and their safety profiles are poorly understood.

**Methods:**

We established two flow cytometry panels for the assessment of two promising leukocyte biomarkers: signal transducer and activator of transcription (STAT) phosphorylation and cytokine receptor expression. We evaluated the first panel, which assesses phosphorylation levels for STAT1, STAT3, and STAT5 after cytokine stimulation, with or without *in vitro* pretreatment with JAKinibs, in 10 healthy donors. We then evaluated the second panel, which assesses cytokine receptor expression on T cells and B cells, in five healthy donors.

**Results:**

Stimulation with interleukin (IL)-2 or IL-7 increased STAT5 phosphorylation in T cells but not in B cells or monocytes. IL-6 stimulation induced STAT3 phosphorylation in monocytes and CD4 T cells and, to a lesser extent, in CD8 T cells, but not in B cells. IL-21 stimulation led to STAT3 phosphorylation in T cells and, to a lesser extent, in B cells, but not in monocytes. Interferon-α stimulation increased STAT1 phosphorylation in all cell types. STAT phosphorylation levels were lower after pretreatment with JAKinibs. A dose–response curve was plotted, confirming the correlation between JAKinib concentration and STAT phosphorylation inhibition. The second panel showed that each cell type displayed a distinct pattern of cytokine receptors expression, and that this pattern might be modified by *in vitro* treatment with JAKinibs.

**Conclusion:**

This preliminary study confirms the utility of flow cytometry for monitoring the biological effects of JAKinibs. Further studies on treated patients are now required to evaluate the clinical value of these two flow cytometry panels.

## Graphical Abstract

**Graphical Abstract:**
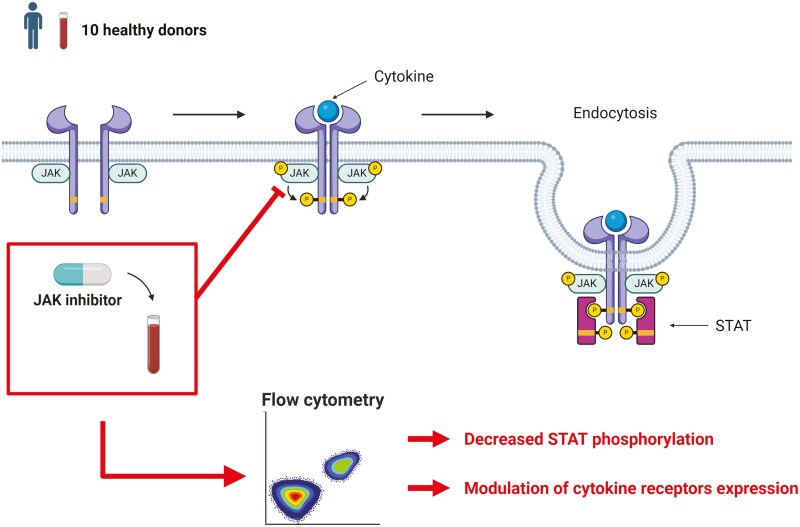

## Introduction

More than 200 germline mutations, somatic mutations, and polymorphisms in *JAK* and *STAT* genes are linked to human diseases, causing either immune dysregulation or malignancies [1]. The Janus kinase (JAK)/signal transducer and activator of transcription (STAT) pathway is also dysfunctional in inflammatory diseases such as atopic dermatitis, rheumatoid arthritis, and inflammatory bowel disease, generally due to an excess of activating cytokines. This excess leads to sustained, uncontrolled inflammation and the development of chronic inflammatory diseases [2–6]. Monoclonal antibodies have greatly improved the treatment of these diseases. However, some patients do not respond to such treatment or develop resistance, with the production of anti-drug antibodies blocking the action of these biotherapies [7]. JAK inhibitors (JAKinibs) block the JAK/STAT pathway by inhibiting the kinase activity of JAK proteins and, therefore, the phosphorylation of STAT proteins. JAKinibs bind to the catalytic site of the JH1 kinase domain of JAK, blocking its kinase activity through competition with ATP (adenosine triphosphate) [8].

JAKinibs are used to treat patients suffering from gain-of-functions mutations of the JAK/STAT pathway [1]. They are also increasingly used to treat chronic inflammatory diseases, but their clinical efficacy is highly variable and their safety profiles are poorly understood [9–11]. Therapeutic drug monitoring may improve the prediction of clinical response, making it possible to optimize drug dose to improve efficacy and limit the adverse effects caused by overdose [6, 12]. There is currently no reliable biomarker for JAKinib monitoring in clinical practice. It has proved difficult to measure JAKinib concentrations in the blood, as these molecules have short half-lives. Furthermore, the concentration of JAKinibs in the blood is not correlated with clinical response [6]. Western blotting can be used to assess the levels of phosphorylated forms of JAK and STAT, but this technique is time-consuming and lacks reproducibility [13]. The inhibition of the JAK/STAT pathway by JAKinibs can be explored by flow cytometry, by measuring the levels of phosphorylated forms of STAT proteins (pSTAT) following cytokine stimulation [9, 14, 15]. Studies performed *in vitro* on whole blood from healthy donors have shown that the potential of JAKinibs to inhibit STAT phosphorylation (expressed as IC_50_) depends on the type of cell and the cytokine used for stimulation [10, 14, 15]. A recent *in vivo* study on 16 rheumatoid arthritis patients showed that tofacitinib significantly decreases cytokine-induced STAT phosphorylation [9]. Activation of the JAK/STAT pathway leads to changes in the expression of certain cytokine receptors. The binding of cytokines to their receptors leads to receptor endocytosis. Under certain circumstances, the receptor can be recycled from the endosomes back to the plasma membrane [16]. For example, it has been shown that stimulation with interleukin (IL)-7 leads to endocytosis of the IL-7 receptor followed by its re-expression at the membrane 3–4 days later [17]. By contrast, the inducible subunit of the IL-2 receptor (CD25) is overexpressed following stimulation of the cell with IL-2 [18]. Following stimulation, the IL-4 receptor is taken up by endocytosis and stored in endosomes, this internalization blocking the subsequent activation of STAT6 [19, 20]. This endocytosis phenomenon is also observed with the IFN-γ receptor, which is internalized in several different cellular compartments, including cytoplasm, mitochondria, and nucleus [21]. Finally, JAKinibs can also decrease the synthesis of pro-inflammatory cytokines. Multiplex immunoassays, such as the Luminex assay, have shown that the *in vitro* treatment of monocytes and T cells with tofacitinib decreases the production of TNF, IL-6, IL-15, and IL-1RA [22]. Tofacitinib can also inhibit the production of IL-4, IL-17, IL-22, and IFN-γ by CD4 T cells from healthy donors following the stimulation of these cells with anti-CD3 antibodies [23]. In phase 2 clinical studies, tofacitinib and filgotinib have been shown to decrease the serum concentrations of pro-inflammatory cytokines, including IL-6 in particular, in patients suffering from rheumatoid arthritis [24, 25].

In this study, we established and compared two whole-blood flow cytometry panels for therapeutic drug monitoring for JAKinibs. The first panel was designed to assess the levels of phosphorylation of STAT1, STAT3, and STAT5 in response to cytokine stimulation, with or without *in vitro* pretreatment with JAKinibs. We also hypothesized that blockade of the JAK/STAT pathway would lead to a variation of membrane expression of cytokine receptors. We therefore designed a second cytometry panel to assess the expression of cytokine receptors on T cells and B cells after *in vitro* incubation with JAKinibs.

## Materials and methods

### Study population

We included 20 healthy donors from the EFS (*Etablisssement Français du Sang*, Saint-Etienne, France) in this study. All donors provided written informed consent for participation in this study, which was approved by the *Centre National Informatique et Liberté* (CNIL number: 1849323) in accordance with the declaration of Helsinki.

### Assessment of STAT phosphorylation

Whole-blood samples (100 µl collected into EDTA for anticoagulation) from healthy donors were either left unstimulated or were stimulated by incubation with 100 ng/ml of IFN-α, IL-6, IL-21, IL-2, or IL-7 (Cell Signaling Technology) for 15 min at 37°C as previously described in the literature [9, 10, 14]. Erythrocytes were lysed and leukocytes were fixed and permeabilized with the PerFix EXPOSE kit (Beckman Coulter) according to the manufacturer’s instructions. They were then incubated with fluorochrome-conjugated antibodies for 15–20 min at room temperature, in the dark. All samples were stained with CD3-PB, CD8-KO, CD14-PC7, and CD19-AAF700 antibodies (Beckman Coulter). Unstimulated cells were also stained with the following anti-pSTAT antibodies (Cell Signaling Technology): pSTAT1-FITC, pSTAT3-APC, and pSTAT5-PE. IFN-α-stimulated cells were stained with a pSTAT1-FITC antibody, whereas cells stimulated with IL-6 or IL-21 were stained with a pSTAT3-APC antibody, and cells stimulated with IL-2 or IL-7 were stained with a pSTAT5-PE antibody. Cells were then washed and resuspended in PerFix EXPOSE kit reagents, according to the manufacturer’s instructions. Antibodies characteristics are detailed in Table 1.

**Table 1.** Summary table for the monoclonal antibodies

Specificity	Clone	Fluorochrome	Purpose/Cell type
CD3	UCHT1	PB	T cells
CD4	13B8.2	APC-AF750	CD4 T cells
CD8	B9.11	KO	CD8 T cells
CD14	RMO52	PC7	Monocytes
CD19	J3-119	APC-AF700	B cells
CD25	B1.49.9	SNv605	IL-2R α chain
CD126	UV4	PC7	IL-6R
CD127	HIL-7R-M21	BV650	IL-7R α chain
CD132	AG184	BV786	Common γ chain receptor
pSTAT1	58D6	FITC	Phosphorylated STAT1
pSTAT3	D3A7	APC	Phosphorylated STAT3
pSTAT5	C71E5	PE	Phosphorylated STAT5

### In vitro blockade with JAK inhibitors

One hour before cytokine stimulation, we incubated 100 µl of EDTA-anticoagulated whole blood from healthy donors with tofacitinib (1 µM), upadacitinib (1 µM), baricitinib (1 µM), or filgotinib (10 µM) (TargetMol) as previously described in the literature [10, 14]. Cells were then left unstimulated or were stimulated with 100 ng/ml of IFN-α, IL-6, IL-21, IL-2, or IL-7 (Cell Signaling Technology) before undergoing the flow cytometry protocol for the assessment of STAT phosphorylation.

### Assessment of cytokine receptor expression

A total of 100 µl of EDTA-anticoagulated whole blood from healthy donors was either incubated with JAK inhibitors (2 or 24 h) or left untreated. Cells were then stained with fluorochrome-conjugated antibodies for 15–20 min at room temperature, in the dark. The following lineage antibodies (Beckman Coulter) were used: CD3-PB, CD4-APC-AF750, CD8-KO, and CD19-APC-AF700. The following antibodies were used to assess the expression of cytokine receptors: CD25-SNv605 (Beckman Coulter), CD132-BV786, CD126-PC7, and CD127-BV650 (BD Biosciences). For each sample, we set up a control tube without antibodies targeting cytokine receptors in parallel. Erythrocytes were lysed by incubation in 1 ml VersaLyse (Beckman Coulter) for 10 min. The remaining cells were washed in 4 ml PBS and centrifuged for 10 min at 300 × g. The resulting cell pellets were suspended in 150 µl of 1% BSA in PBS. Antibodies characteristics are detailed in Table 1.

### Flow cytometry analysis

Samples were analysed on a DxFLEX B5-R3-V5 cytometer (3 lasers, 13 detectors) with CytExpert software (Beckman Coulter). We compensated for spectral overlaps with the VersaComp Antibody Capture kit (Beckman Coulter). Cytometer settings were checked and standardized daily with DxFLEX Daily QC Fluorospheres (Beckman Coulter). We assessed pSTAT levels by calculating the median fluorescence intensity (MdFI) ratio between stimulated cells and unstimulated cells. We assessed the expression of cytokine receptors by calculating MdFI ratios between the fully stained tube and the control tube for each receptor.

### Statistical analysis

Statistical analyses were performed with GraphPad Prism software. The statistical significance was assessed using Friedman tests followed by Dunn’s post-hoc tests. When data were missing, Friedman tests were replaced by mixed effects models followed by Tukey’s post-hoc tests (for the expression of CD127 in CD4 T cells and in B cells). A *P*-value < .05 was considered statistically significant.

## Results

### Cytokine stimulation increases STAT phosphorylation in vitro

**Figure 1.**
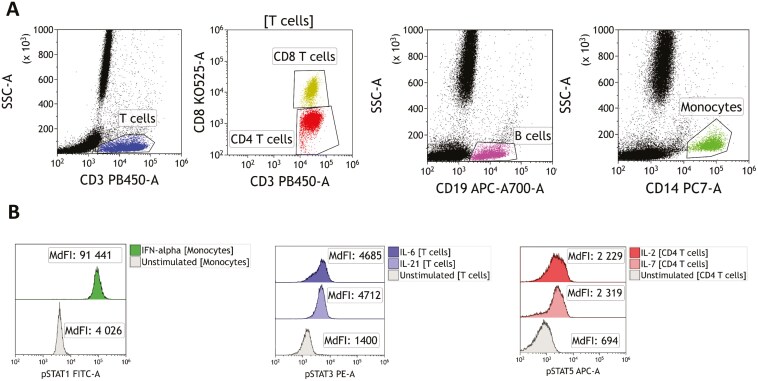
Gating strategy for the assessment of STAT phosphorylation. (A) Gating of leukocyte subpopulations. T cells are defined as CD3 positive cells. Among T cells, CD4 T cells are defined as CD3 positive and CD8 negative cells and CD8 T cells as CD3 and CD8 positive cells. B cells are defined as CD19 positive cells and monocytes as CD14 positive cells. (B) Representative overlay histograms of STAT1, STAT3, and STAT5 phosphorylation in unstimulated and stimulated monocytes, total T cells, and CD4 T cells.

**Figure 2.**
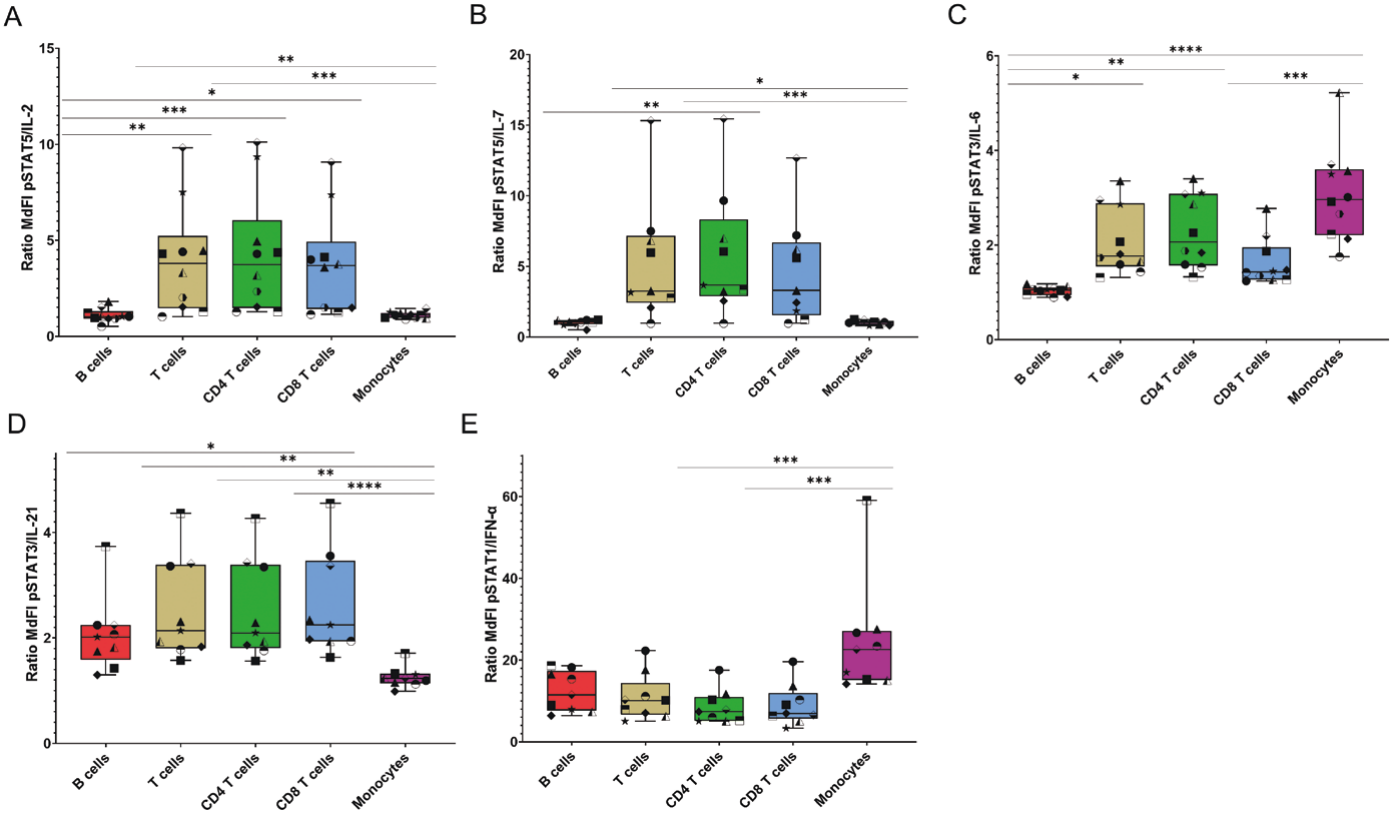
Cytokine stimulation increases STAT phosphorylation *in vitro*. Ratios of the MdFI for pSTAT in stimulated cells to the MdFI for pSTAT in unstimulated cells from healthy donors. (A) pSTAT5/IL-2, (B) pSTAT5/IL-7, (C) pSTAT3/IL-6, (D) pSTAT3/IL-21, and (E) pSTAT1/IFN-α. Tukey box plots represent the first quartile (lower side of the box), the median (middle line of the box), and the third quartile (upper side of the box). The interquartile range is represented by the distance between the first and the third quartiles. Whiskers represent the minimum and the maximum. Each symbol represents a healthy donor. *n* = 10. The statistical significance was assessed using Friedman tests followed by Dunn’s post-hoc tests. *P < .05, **P < .01, ***P < .001, ****P < .0001.

We assessed constitutive and cytokine-induced STAT phosphorylation by flow cytometry in 10 healthy donors. The gating strategy is depicted in Fig. 1. STAT phosphorylation was considered significant when MdFIs ratios between cytokine-stimulated and unstimulated cells were above 1.5. We found that stimulation with IL-2 or IL-7 increased STAT5 phosphorylation in T cells (mean MdFI ratio IL-2: 4, range: 1–9.8 / IL-7: 5.3, 1–15.3) but not in B cells (IL-2: 1.1, 0.5–1.8 / IL-7: 1, 0.5–1.2) or monocytes (IL-2: 1.1, 0.9–1.5 / IL-7: 1, 0.8–1.2) (Fig. 2A and B). IL-6 stimulation led to STAT3 phosphorylation in monocytes (3.1, 1.8–5.2) and CD4 T cells (2.3, 1.3–3.4) and, to a lesser extent, in CD8 T cells (1.6, 1.2–2.8), but not in B cells (1, 0.9–1.2) (Fig. 2C). IL-21 stimulation led to STAT3 phosphorylation in T cells (2.5, 1.6–4.4) and, to a lesser extent, B cells (2.1, 1.3–3.7), but not monocytes (1.3, 1–1.7) (Fig. 2D). IFN-α stimulation strongly increased STAT1 phosphorylation in all cell types, with mean MdFI ratios ranging from 8.5 (CD4 T cells) to 24.5 (monocytes) (Fig. 2E).

### JAK inhibitors prevent STAT phosphorylation in a dose-dependent manner

**Figure 3.**
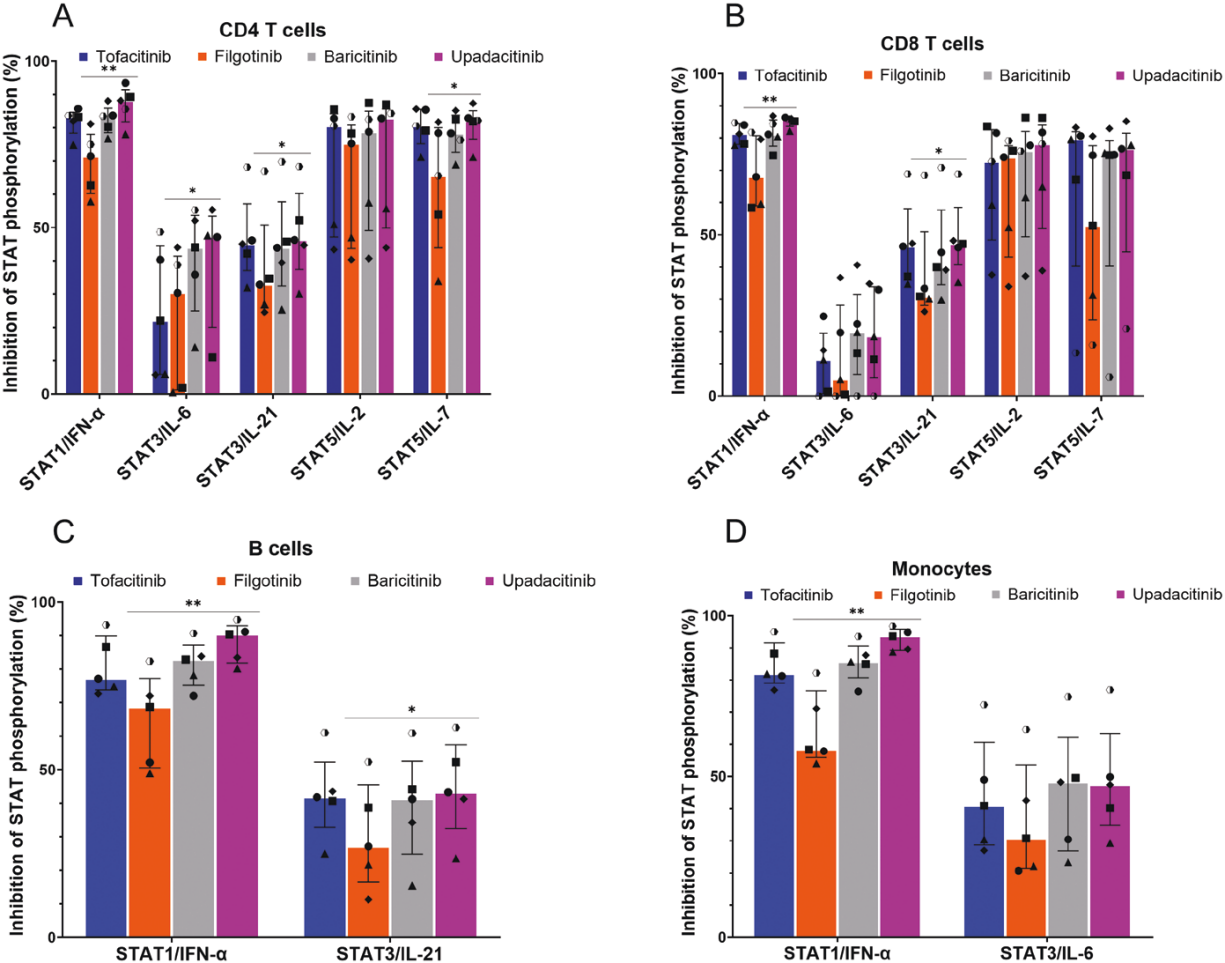
JAK inhibitors prevent cytokine-induced STAT phosphorylation. Inhibition of STAT phosphorylation by JAK inhibitors in (A) CD4 T cells, (B) CD8 T cells, (C) B cells, and (D) monocytes from healthy donors. Bar plots represent the median value of STAT phosphorylation inhibition (in %). Whiskers represent the interquartile range. Each symbol represents a healthy donor. *n* = 5. The statistical significance was assessed using Friedman tests followed by Dunn’s post-hoc tests. *P < .05, **P < .01.

We assessed the ability of JAK inhibitors to prevent cytokine-induced STAT phosphorylation in five healthy donors. Overall, STAT phosphorylation levels were lower in the presence of JAK inhibitors (Fig. 3). However, the percent inhibition varied greatly, depending on the cytokine/STAT pathway. Highest inhibition was observed for STAT1/IFN-α, regardless of cell type. For STAT1/IFN-α, MdFI ratios of uninhibited cells ranged from 8 to 25.2 compared to inhibited cells whose ratios ranged from 1 to 7.4. For STAT3/IL-6, they ranged from 1.4 to 2.6 (uninhibited cells) and from 1.1 to 1.5 (inhibited cells). For STAT3/IL-21, they ranged from 2 to 2.3 (uninhibited cells) and from 1 to 1.3 (inhibited cells). For STAT5/IL-2, they ranged from 1.1 to 3.6 (uninhibited cells) and from 0.8 to 1.2 (inhibited cells). For STAT5/IL-7, they ranged from 1.2 to 4.6 (uninhibited cells) and from 0.8 to 1.5 (inhibited cells). Interestingly, T cells displayed lower inhibition for STAT3 compared to STAT1 and STAT5 (Fig. 3A and B). We also observed differences between the four JAK inhibitors, although they generally induced inhibition levels of similar magnitudes. Upadacitinib exhibited the highest inhibition rate, with MdFI ratios ranging from 1 to 1.4, while filgotinib seemed to be the less potent (MdFI ratios ranging from 1 to 7.4). Tofacitinib and baricitinib appeared to be in-between, with MdFI ratios ranging from 0.8 to 3 for tofacitinib, and from 0.8 to 3.1 for baricitinib. However, statistical significances were only observed between upadacitinib and filgotinib. In all cell types, STAT1 phosphorylation inhibition was greater with upadacitinib than with filgotinib (*P* < .01). In CD4 T cells, STAT3 (*P* < .05) and STAT5/IL-7 (*P* < .05) phosphorylation were also more effectively inhibited by upadacitinib compared to filgotinib (Fig. 3A). These differences were also observed in CD8 T cells but statistical significance was absent for STAT3/IL-6 and STAT5/IL-7 (Fig. 3B). In B cells, differences were significant for STAT3/IL-21 (*P* < .05) (Fig. 3C).

**Figure 4.**
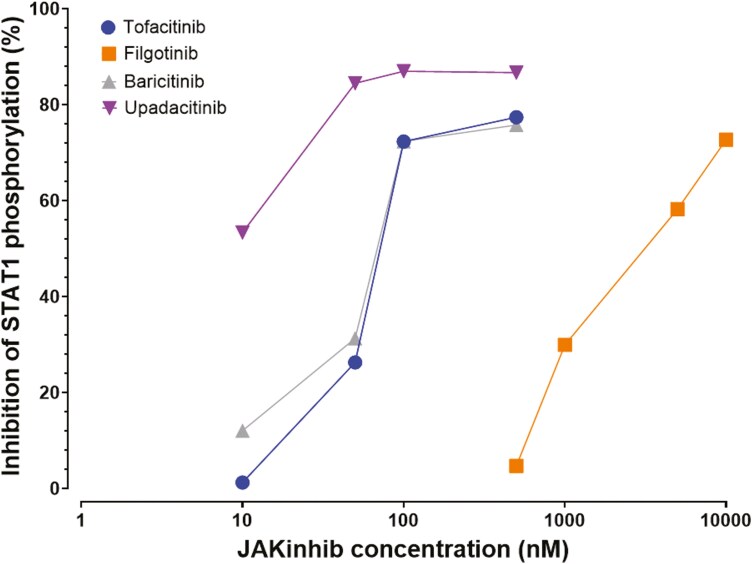
JAK inhibitors prevent STAT phosphorylation in a dose-dependent manner. Inhibition of STAT1 phosphorylation as a function of JAK inhibitor concentration. The mean percent inhibition was calculated by pooling the data from all leukocyte subpopulations (T cells, B cells, and monocytes). *n=*1.

We then investigated the dose–response relationship between JAK inhibitor concentration and STAT phosphorylation. We assessed the inhibition of STAT1 phosphorylation, as IFN-α stimulation caused a substantial increase in the phosphorylation of this molecule, facilitating the detection of very low levels of phosphorylation inhibition due to JAK inhibitors. The percent inhibition was positively correlated with concentration for all four JAK inhibitors tested (Fig. 4). The curves for baricitinib and tofacitinib overlapped, whereas higher concentrations of filgotinib were required to achieve the same degree of STAT1 phosphorylation inhibition. Interestingly, upadacitinib appeared to be the most potent drug as 75% inhibition was achieved with lower concentrations of this molecule.

### Cytokine receptor expression after treatment with JAK inhibitors

**Figure 5.**
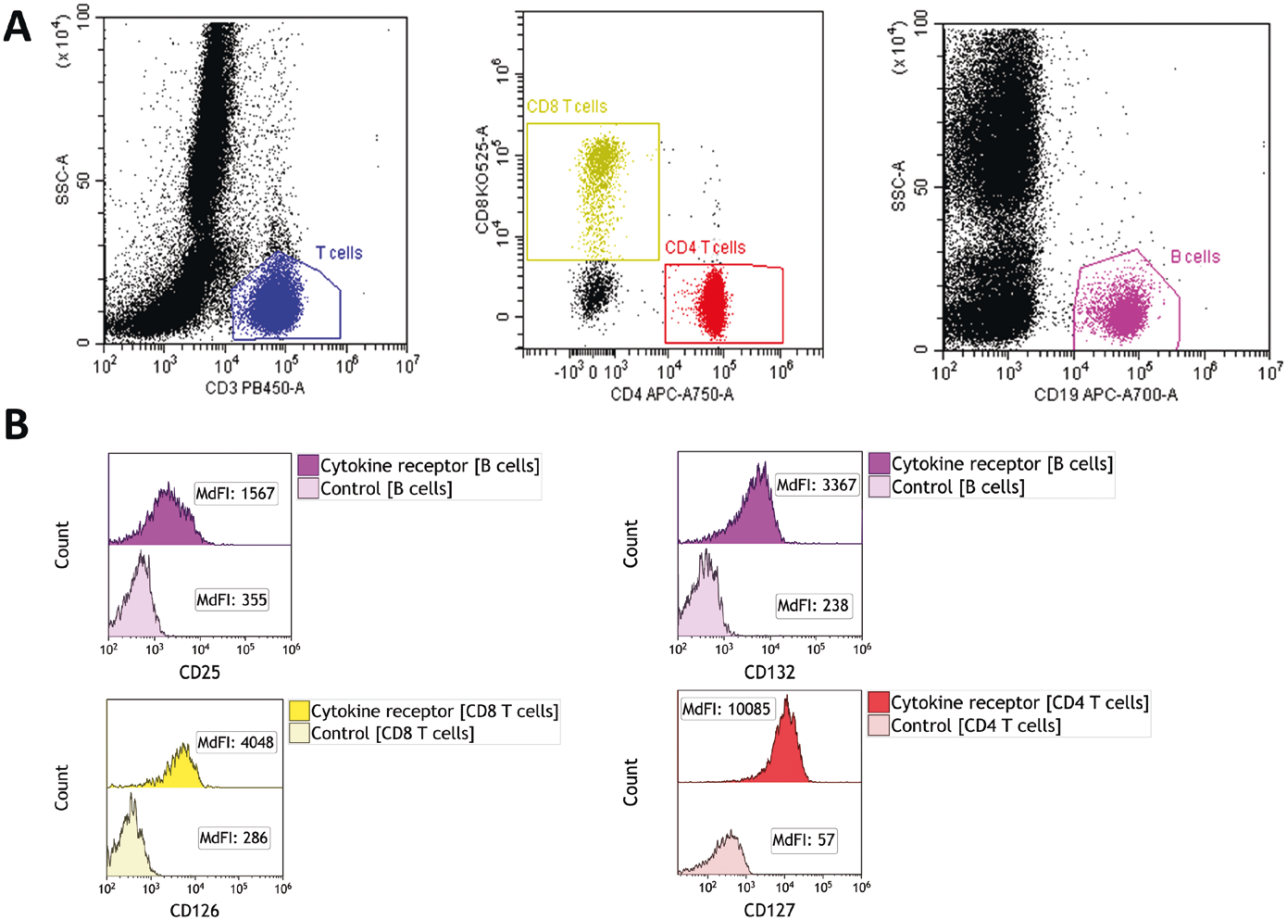
Gating strategy for the assessment of cytokine receptor expression. (A) Gating of leukocyte subpopulations. T cells are defined as CD3 positive cells. Among T cells, CD4 T cells are defined as CD3 and CD4 positive cells and CD8 T cells as CD3 and CD8 positive cells. B cells are defined as CD19 positive cells. (B) Representative overlay histograms of cytokine receptors expression by B cells (CD25 and CD132), CD8 T cells (CD126), and CD4 T cells (CD127).

**Figure 6.**
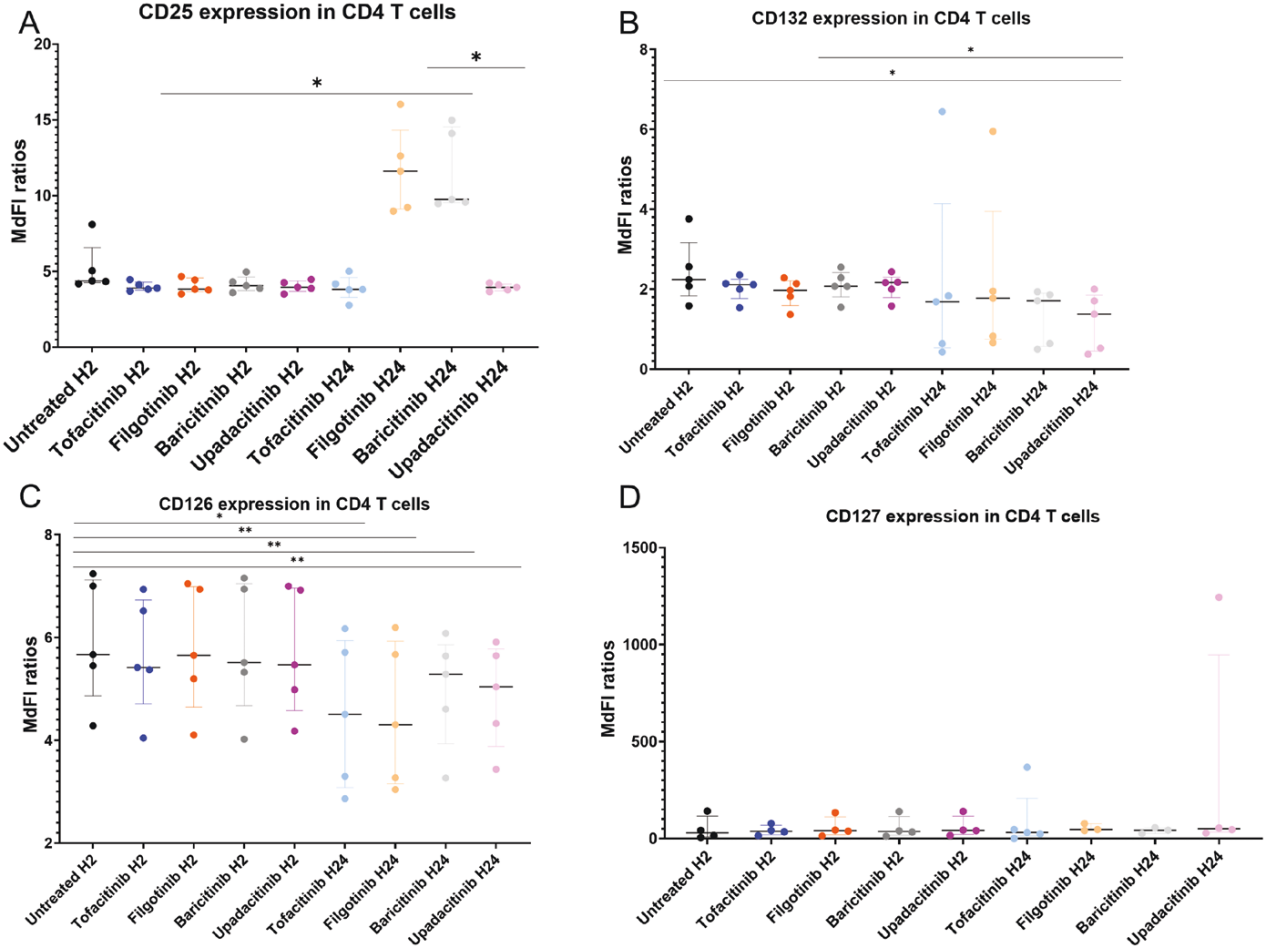
Expression of cytokine receptors in CD4 T cells. MdFI ratios between fully stained tubes and control tubes for (A) CD25, (B) CD132, (C) CD126, and (D) CD127. Dot plots represent the median and the interquartile ranges. *n* = 5. The statistical significance was assessed using Friedman tests followed by Dunn’s post-hoc tests. When data were missing (CD127), Friedman tests were replaced by mixed effects models followed by Tukey’s post-hoc tests. *P < .05, **P < .01.

**Figure 7.**
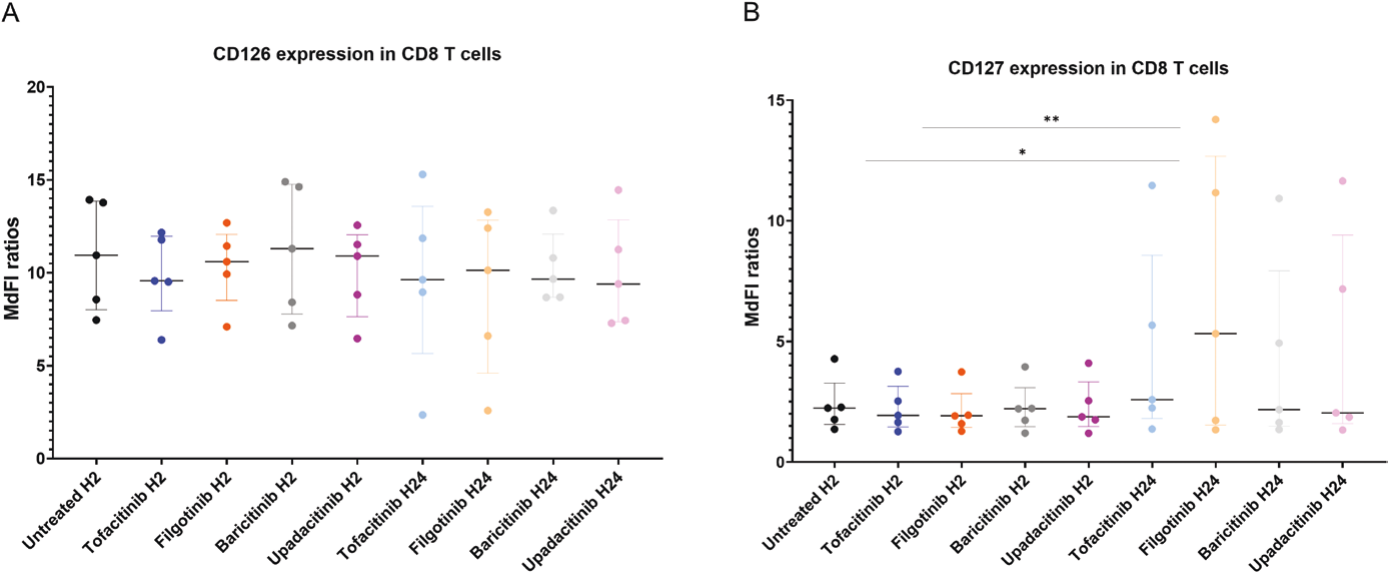
Expression of cytokine receptors in CD8 T cells. MdFI ratios between fully stained tubes and control tubes for (A) CD126 and (B) CD127. Dot plots represent the median and the interquartile ranges. *n* = 5. The statistical significance was assessed using Friedman tests followed by Dunn’s post-hoc tests. *P < .05, **P < .01.

**Figure 8.**
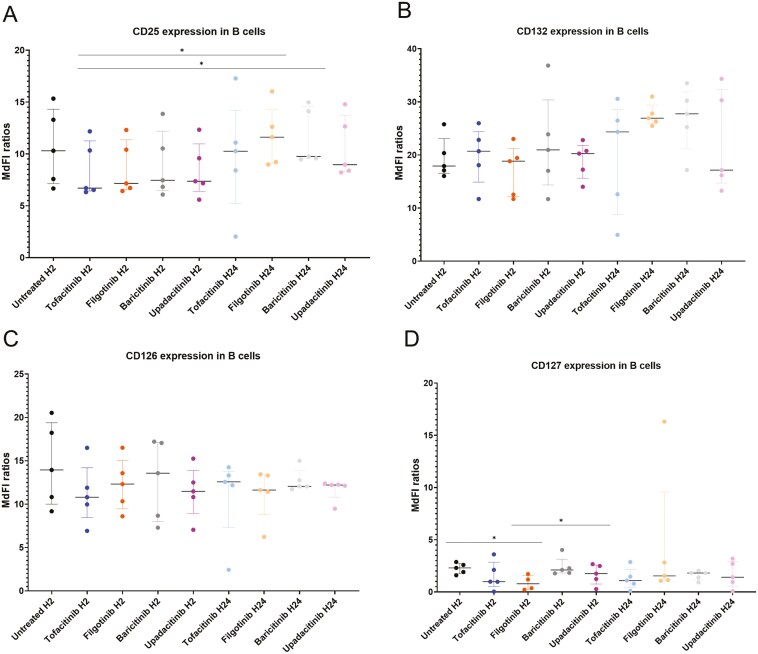
Expression of cytokine receptors in B cells. MdFI ratios between fully stained tubes and control tubes for (A) CD25, (B) CD132, (C) CD126, and (D) CD127. Dot plots represent the median and the interquartile ranges. *n* = 5. The statistical significance was assessed using Friedman tests followed by Dunn’s post-hoc tests. When data were missing (CD127), Friedman tests were replaced by mixed effects models followed by Tukey’s post-hoc tests. *P < .05.

We assessed the expression of cytokine receptors in five healthy donors, after an *in vitro* incubation with JAKinibs (2 and 24 h) and in the absence of JAKinib treatment. The gating strategy is depicted in Fig. 5. In CD4 T cells, we detected a significant increase of CD25 expression after 24-h treatment with filgotinib or baricitinib (*P* < .05) (Fig. 6A). On the opposite, CD132 expression was decreased after 24-h treatment with baricitinib or upadacitinib (*P* < .05 for upadacitinib only) (Fig. 6B) and CD126 expression was decreased after 24-h treatment with any of the JAKinib (*P* < .05 for tofacitinib, *P* < .01 for the three other JAKinibs) (Fig. 6C). No significant differences were observed for CD127 expression (Fig. 6D). In CD8 T cells, no statistical differences were identified for CD126 expression while slight increases of CD127 expression were observed after 24-h treatments with JAKinibs (Fig. 7A and B). In B cells, CD25 expression was slightly increased after 24-h treatment with filgotinib or baricitinib (*P* < .05), while CD127 expression was decreased after 2-h treatment with filgotinib or upadacitinib (*P* < .05). No significant differences were observed for CD132 and CD126 expression (Fig. 8A–D).

## Discussion

We established a whole-blood flow cytometry protocol for assessing STAT phosphorylation in healthy donors. Using this protocol, we showed that STAT phosphorylation significantly increased following cytokine stimulation. The strongest signals obtained were those for pSTAT1 following IFN-α stimulation. Stimulation with IL-2 or IL-7 led to an increase in STAT5 phosphorylation in T cells but not in B cells or in monocytes, which was expected as receptors of IL-2 and IL-7 are mainly expressed on T cells [26, 27]. Both IL-6 and IL-21 induced STAT3 phosphorylation, but in different cell types: CD4 T cells and monocytes for IL-6, T cells and B cells for IL-21.

Treatment with JAKinibs *in vitro* resulted in lower levels of STAT phosphorylation. Percent inhibition varied considerably between cytokine/STAT pathways and cell types. In T cells, STAT3 phosphorylation inhibition was lower compared to STAT1 and STAT5. This can be explained by the fact that T cells stimulation by IL-6 or IL-21 result in lesser STAT phosphorylation (mean ratio of 2 for IL-6 and 2.2 for IL-21) compared to IL-2 (3), IL-7 (3.7), and IFN-α (13.5). Indeed, the higher STAT is phosphorylated after cytokine stimulation, the easier is to detect any slight inhibition caused by JAK inhibitors. Percent variation also varied according to the type of JAK inhibitors, as upadacitinib was consistently the most powerful drug while filgotinib was consistently the weakest. These discrepancies between upadacitinib and filgotinib cannot be linked to JAK specificities as upadacitinib and filgotinib both selectively inhibit JAK1. A dose–response curve was plotted for STAT1/IFN-α in one healthy donor, confirming the correlation between JAKinib concentration *in vitro* and percent inhibition of STAT. Filgotinib was once again the least potent JAKinib, whereas upadacitinib was the most potent. These results require confirmation in a larger cohort as the inhibition experiments were performed on only five healthy donors and the dose–response curves were obtained with blood from a single healthy donor. Dose–response curves should also be plotted for other cytokine/STAT pathways. Indeed, Traves *et al*. reported that the inhibition of STAT phosphorylation in healthy donors was 7–11 times greater for IL-6/STAT1 than for IL-6/STAT3, regardless of cell type or JAKinib [14].

Our results are consistent with published findings as other studies have also identified that filgotinib was the least potent JAKinib. Using whole-blood flow cytometry techniques, Dowty *et al*. and Traves *et al*. showed that filgotinib had an IC_50_ 10–100 times higher than those of other JAKinibs, regardless of cell type or cytokine/STAT pathway [10, 14]. Another recent study on PBMCs from healthy donors also confirmed that the levels of inhibition achieved with JAKinibs depended on the cytokine/STAT pathway, which is not surprising, as each JAKinib has different specificities for different JAK proteins [15]. However, some of our results conflict with those of other studies. Traves *et al*. showed that there was a three-fold difference in the level of inhibition of STAT1 phosphorylation between CD4 T cells and monocytes, regardless of the JAKinib considered [14]. We found no such difference, but our study included only five healthy donors whereas that of Traves *et al.* included 10.

The application of this protocol to a larger number of healthy donors would help to highlight more differences and would strengthen our observations. It would also make it possible to establish—for each cell type, JAKinib, and cytokine/STAT pathway—a mean pSTAT fluorescence ratio for use as a reference value for healthy donors. Furthermore, it might be possible to improve this protocol by testing other cytokine/STAT pathways and different concentrations of cytokines.

Finally, this flow cytometry protocol requires testing on samples from patients treated with JAKinibs in order to confirm our results on healthy donors treated *in vitro*. Palmroth *et al*. used whole-blood flow cytometry to assess the phosphorylation of STAT proteins after the stimulation, with cytokines, of samples from 16 rheumatoid arthritis patients on tofacitinib treatment [9]. The results of their *in vivo* study conflicted with those of the *in vitro* studies cited above [10, 14, 15]. Indeed, the authors showed that the percent inhibition achieved with tofacitinib was lower *in vivo*, suggesting that *in vitro* studies may overestimate the inhibitory potential of this drug. Overall, these findings demonstrate the need for more *in vivo* studies in patients suffering from different chronic inflammatory diseases (rheumatologic, gastroenterologic, and dermatologic diseases) treated with different JAK inhibitors. The ultimate goal is to evaluate this protocol’s capacity to discriminate between responders and non-responders and to predict the occurrence of side effects and losses of response. It would also be of interest to validate this protocol on samples from patients suffering from gain-of-function (GOF) mutations of the JAK/STAT pathway. In such cases, our protocol is expected to confirm the enhancement of STAT phosphorylation after *in vitro* cytokine stimulation. Indeed, Albuquerque *et al*. have shown that patients carrying GOF mutations of STAT1 displayed increased STAT1 phosphorylation after IFN-α stimulation compared to healthy donors [28].

We also investigated the expression of cytokine receptors on T cells and B cells, with or without *in vitro* JAKinib treatment. Several studies have shown that the binding of cytokines to their receptors induces the endocytosis or the overexpression of the receptor [16, 18–21, 29]. We hypothesized that JAKinibs, by blocking the cytokine/STAT pathway, alter the expression of cytokine receptors on the leukocyte membrane. Surprisingly, CD25 and CD132 expression could not be quantified in CD8 T cells, although IL-2 induced significant STAT5 phosphorylation. This could be explained by a lack of sensitivity of our flow cytometry protocol making it difficult to detect and quantify cytokine receptors with low surface densities. In B cells, CD25, CD127, and CD132 could be quantified although IL-2 and IL-7 did not induce STAT5 phosphorylation. The lack of response to a cytokine stimulus despite the presence of high levels of its receptor may be explained by inadequate cytokine stimulation, due to a cytokine concentration that is too low or a suboptimal incubation time. When treated with filgotinib or baricitinib for 24 h, CD4 T cells displayed increased CD25 expression. However, CD132 expression was decreased after treatment with baricitinib or upadacitinib. The discrepancy for baricitinib is difficult to explain as CD25 and CD132 constitute the IL-2 receptor, thus we expected them to display similar expression patterns. Of note, CD126 expression was decreased after 24-h treatment with any of the JAKinib, confirming that each cytokine receptor exhibits a singular expression pattern after JAKinib treatment. JAKinibs do not seem to modulate CD127 expression on T cells. We have observed significant increases in CD127 expression in CD8 T cells, but they were most likely caused by outliers. CD127 and CD132 constitute the IL-7 receptor but we were unable to highlight similar expression patterns. In B cells, significant differences in CD25 and CD127 expression were observed after JAKinib treatment, but they are difficult to interpret as IL-2 and IL-7 failed to induce STAT phosphorylation in B cells.

Altogether, these results fail to show a clear effect of *in vitro* JAKinibs treatment on cytokine receptor expression. Perhaps incubation time with JAKinibs should be longer, as cytokine receptors expression can fluctuate up to 10 days after lymphocyte activation [14]. Studies in a larger number of healthy donors are warranted to achieve greater statistical robustness and to conclude on the relevancy of measuring cytokine receptors expression for monitoring JAKinib effects. Furthermore, it would be of interest to study cytokine receptors expression in patients suffering from genetic defects of the JAK/STAT pathway.

This preliminary work confirms the utility of flow cytometry for monitoring the biological effects of JAKinibs through the determination of pSTAT levels after cytokine stimulation. Studies including healthy donors and treated patients responding or not responding to JAKinibs are now required. Ideally, a longitudinal study should be performed, with patients undergoing sampling before the initiation of JAKinib treatment and regularly thereafter. This protocol could easily be implemented in a hospital laboratory because flow cytometry is a rapid, reproducible technique. It would also be interesting to assess the expression of cytokine receptors by flow cytometry in patients treated with JAKinibs in order to evaluate the clinical value of this other protocol.

## Data Availability

The data underlying this article will be shared upon reasonable request to the corresponding author.
